# Synergistic Enhancement of Paramylon Production in Edible Microalga *Euglena gracilis* via Ethanol-Guaiacol Co-Regulation

**DOI:** 10.3390/foods14142457

**Published:** 2025-07-12

**Authors:** Xinyi Yan, Hao Xu, Zhengfei Yang, Yongqi Yin, Weiming Fang, Minato Wakisaka, Jiangyu Zhu

**Affiliations:** 1 School of Food Science and Engineering, Yangzhou University, Yangzhou 225127, China; 222403230@stu.yzu.edu.cn (X.Y.); 243601119@stu.yzu.edu.cn (H.X.); yzf@yzu.edu.cn (Z.Y.); yqyin@yzu.edu.cn (Y.Y.); wmfang@yzu.edu.cn (W.F.); 2 Food Study Centre, Fukuoka Women’s University, 1-1-1 Kasumigaoka, Fukuoka 813-8529, Japan

**Keywords:** *Euglena gracilis*, guaiacol, photosynthetic pigment, paramylon

## Abstract

Biomass-derived growth stimulants are widely recognized as green and economical solutions that can significantly enhance microalgae culture efficiency and optimize the biomanufacturing process of target products. In this paper, we investigated the effect of ethanol synergized with guaiacol (GA) on biomass and β-1,3 glucan accumulation in edible microalgae, namely *Euglena gracilis*. The ethanol-induced mixotrophic mode significantly increased biomass and paramylon production by 12.68 and 6.43 times, respectively, compared to the autotrophic control group. GA further exerted toxic excitatory effects (hormesis) on top of ethanol mixotrophic nutrition. At the optimal concentration of 10 mg·L^−1^ GA, chlorophyll a, carotenoids, and paramylon production increased by 8.96%, 11.75%, and 16.67%, respectively, compared to the ethanol-treated group. However, at higher concentrations, the biomass and paramylon yield decreased significantly. This study not only establishes an effective combinatorial strategy for enhancing paramylon biosynthesis but also provides novel insights into the hormesis mechanism of phenolic compounds in microalgae cultivation. The developed approach demonstrates promising potential for sustainable production of high-value algal metabolites while reducing cultivation costs, which could significantly advance the commercialization of microalgae-based biorefineries in food and pharmaceutical industries.

## 1. Introduction

Against the backdrop of the rapid development of sustainable biomanufacturing technologies, microalgae, with their unique carbon sequestration capacity, efficient photosynthetic metabolism system, and non-cultivated land dependence, have become a strategic bio-resource with outstanding potentials for multi-disciplinary applications. Compared with traditional oil crops (e.g., soybean, corn, etc.), microalgae can synthesize a variety of high-value metabolites without the need to occupy agricultural land. This characteristic makes microalgae show unique advantages in the industrial chain of functional food additives, bioactive substance synthesis, animal feed improvement, and clean energy development [1,2]. Of particular interest is the flagellated microalga *Euglena gracilis*, which exhibits distinct traits including phototactic motility (mediated by an eyespot), absence of a rigid cell wall, and the unique capacity to synthesize paramylon—a linear β-1,3-glucan polymer with documented prebiotic and immunostimulatory properties [3,4]. Paramylon is increasingly recognized as a high-value functional biopolymer due to its exceptional water insolubility, resistance to mammalian digestive enzymes, and ability to modulate immune responses (e.g., activation of macrophage phagocytosis and cytokine production) [5]. These attributes make it a promising candidate for nutraceuticals (e.g., gut health supplements), immune-boosting therapeutics, and biodegradable materials [4,6]. *E. gracilis* leverages its versatile metabolic modes (photoautotrophic, mixotrophic, and heterotrophic) to dynamically redirect carbon flux toward target metabolites such as paramylon and medium-/long-chain fatty acids [3]. In recent years, new strategies to optimize the allocation of metabolic fluxes in microalgae through the introduction of exogenous regulatory factors have attracted much attention in the field of metabolic engineering, among which the regulatory potential of lignin-derived phenolic compounds (e.g., guaiacol) as natural growth elicitors has gained significant research interest, with recent studies elucidating their roles in redirecting algal metabolic fluxes and enhancing stress resilience [7,8,9].

Guaiacol (GA; 2-methoxyphenol) was selected for this study due to its prominence as a lignin depolymerization monomer, and its structural suitability for transmembrane diffusion. Prior screening of lignin-derived phenolics identified syringic acid, p-coumaric acid, etc., as particularly effective for enhancing paramylon yield in *E. gracilis* [8,9]. However, systematic studies on the metabolic regulatory effects of GA on *E. gracilis* remain scarce, and its bioavailability, transmembrane transport mechanisms, and metabolic redirection patterns in this alga are as yet undefined—particularly due to the intrinsically low water solubility of GA, which severely restricts cellular uptake. To overcome this limitation, we synergistically combined ethanol’s dual functionality: as a carbon source for mixotrophic growth at concentrations ≤3%, which significantly enhances biomass production [10], and as a solubilizing agent to improve GA’s aqueous dispersion and bioavailability. This synergistic strategy facilitates efficient intracellular delivery of GA while supporting cellular energy metabolism.

Therefore, this study seeks to develop a GA-ethanol co-regulation system by assessing growth profiles (cell density and biomass), photosynthetic pigment levels (chlorophyll a/b and carotenoids), and the production of the high-value compound paramylon. Notably, this work establishes the first demonstration of guaiacol-mediated hormesis in *E. gracilis* under ethanol-induced mixotrophy, revealing its dose-dependent dual effects on paramylon biosynthesis. This approach offers an economically feasible and eco-friendly solution to support the goal of carbon neutrality.

## 2. Materials and Methods

### 2.1. Chemical, Reagent and Algal Strain

Analytical-grade guaiacol (2-methoxyphenol, GA, Product No. G5502, ≥98% purity) was purchased from Sigma-Aldrich (St. Louis, MO, USA). Molecular-biology-grade absolute ethanol (Product No. FJ057-00456, ≥99.5% purity) was obtained from Wako Chemicals (Osaka, Japan). Modified Cramer–Myers (CM) media components [11], including macronutrients, micronutrients, and vitamins, were acquired from Nacalai Tesque, Inc. (Kyoto, Japan). The basal medium contained the following components (mg·L^−1^): (NH_4_)_2_HPO_4_, 1000; KH_2_PO_4_, 1000; MgSO_4_·7H_2_O, 200; CaCl_2_·2H_2_O, 20; FeSO_4_·7H_2_O, 3; MnCl_2_·4H_2_O, 1.8; CoSO_4_·7H_2_O, 1.5; ZnSO_4_·7H_2_O, 0.4; Na_2_MoO_4_·2H_2_O, 0.2; CuSO_4_·5H_2_O, 0.02; Vitamin B12, 0.0005; Thiamine HCl, 0.1. All other reagents used were of highest commercially available purity grade. The freshwater green microalga *E. gracilis* (NIES-2145) was procured from the microalgae culture collection at National Institute for Environmental Studies (NIES), Japan. The strain was maintained in CM liquid media under standard culture conditions of 25 °C temperature, 120 μmol·m^−2^·s^−1^ light intensity, and 12:12 h light/dark photoperiod with monthly subculture.

### 2.2. Preparation of Stock Solutions and Experimental Design

A concentrated guaiacol (GA) stock solution (2000 mg·L^−1^) was prepared by dissolving pure GA in absolute ethanol and CM medium, achieving a final ethanol concentration of 2% (*v*/*v*). Concurrently, a separate ethanol stock solution (2% *v*/*v* in CM medium) was prepared. Both stock solutions were filter-sterilized using 0.2 μm polyethersulfone membrane filters (Sartorius Stedim Biotech, Göttingen, Germany). Experimental cultures were established in 300 mL Erlenmeyer flasks by combining appropriate volumes of the GA stock solution, ethanol stock solution, fresh CM medium, and mid-exponential phase algal inoculum to achieve a total volume of 100 mL per flask. This protocol yielded final GA concentrations of 10, 50, 200, and 1000 mg·L^−1^ while maintaining a consistent ethanol concentration of 1% (*v*/*v*) in all ethanol-supplemented groups. The initial cell density was standardized to 6 × 10^4^ cells·mL^−1^ across all treatments. Specifically, the control group (CK) contained no GA or ethanol; the solvent control group (CE) contained 1% ethanol without GA; and GA treatment groups (designated CE-G10, CE-G50, CE-G200, CE-G1000) contained both 1% ethanol and their respective GA concentrations. All flasks were incubated at 25 °C under 120 μmol photons·m^−2^·s^−1^ illumination with a 12:12 h light/dark photoperiod, with manual agitation twice daily. The experiment was performed in triplicate for each condition.

### 2.3. Cell Growth Analysis

The growth profile of *E. gracilis* under varying GA concentrations was monitored by recording cell count, optical density (OD), and cell dry weight (DW). Cell counting was done in triplicates using a hemocytometer (Paul Marienfeld, Germany). Optical density at a wavelength of 680 nm (OD680nm) was measured spectrophotometrically in a UV-2450 system (Shimdazu, Kyoto, Japan) after adequately diluting samples. For DW, 15 mL culture was vacuum filtered through pre-dried and pre-weighed 0.45 μm GF/C filter paper (Whatman). The filtered biomass was washed three times with sterile distilled water, and the filter paper was dried at 105 °C overnight before being weighed using an analytical balance (Mettler Toledo, Greifensee, Switzerland). Specific growth rates were calculated and compared between control and treatments. The pH variations were also recorded using a digital pH meter (Hanna Instruments, Woonsocket, RI, USA).

### 2.4. GA Quantification

Residual GA in culture supernatant after removing cells was estimated by recording absorbance at 275 nm using UV-1800 spectrophotometer (Shimdazu, Kyoto, Japan) based on a standard curve prepared identically with known GA concentrations [12]. Full spectral scans (200–350 nm) were additionally performed for all GA treatment groups at Day 0 and Day 15 to detect oxidative byproducts (e.g., quinones) that alter spectral profiles. The cell-free filtrate was obtained by filtering 1 mL sample through a 0.23 μm PES membrane syringe filter.

### 2.5. Photosynthetic Pigments Analysis

Five-milliliter algal culture samples were centrifuged at 5000× *g* for 5 min at 4 °C. The cell pellets were resuspended in 5 mL of ice-cold 80% (*v*/*v*) acetone by vertexing for 1 min and incubated overnight at 4 °C in dark for complete pigment extraction. The crude extracts were clarified via centrifugation at 10,000× *g* for 10 min. The clear supernatants were analyzed on a Shimadzu UV-2450 spectrophotometer (Shimdazu, Kyoto, Japan) and recordings taken at 470, 646, and 663 nm. Chlorophyll a (Chl_a_), chlorophyll b (Chl_b_), and total carotenoid (C_x+c_) contents were determined using the standard spectrophotometric equations of Lichtenthaler and Wellburn [13].

### 2.6. Paramylon Content and Yield

Paramylon content (% of dry basis) and volumetric yield (g·L^−1^) were determined gravimetrically [8]. Briefly, 30 mL of culture sample was harvested via centrifugation at 4000× *g* for 10 min. The cell pellet was washed twice with distilled water and recentrifuged. The supernatant was discarded, and the cell pellet was lyophilized. To remove esterified compounds, the lyophilized biomass was mixed with 100 mL propanol and incubated at 45 °C for 12 h in the dark with intermittent shaking. The mixture was centrifuged at 4000× *g* for 10 min, and the solvent fraction was discarded. The pellet was resuspended in 30 mL of 50 mM sodium acetate buffer (pH 5.5). Cell disruption was performed by ultrasonication at 600 W output power for 10 cycles (60 s pulses with 60 s breaks) using a probe ultrasonicator (BEM-650A, Bueno Biotech, Nanjing, China). The lysate was centrifuged at 9000× *g* for 30 min, and the pellet containing paramylon particles was collected. This pellet was washed twice with distilled water followed by centrifugation (9000× *g*, 30 min). The paramylon particles were resuspended in 30 mL of 1% (*w*/*v*) SDS solution, incubated at 100 °C for 30 min to remove membrane contaminants, cooled to room temperature, and centrifuged (9000× *g*, 30 min). The purified paramylon pellet was washed twice with distilled water, dried overnight at 60 °C, and weighed. Paramylon yield (g·L^−1^) and content (% dry basis) were calculated as:

Paramylon yield (g·L^−1^) = W_p_ ÷ V_p_ (1)

Paramylon content (% dry basis) = Paramylon yield ÷ DW × 100(2)

where W_p_ (g), V_p_ (L), and DW (g·L^−1^) are the weight of purified paramylon pellet, the volume of harvested algal solution, and dry weight, respectively.

### 2.7. Statistical Analysis

All analytical assays were performed in triplicates for each concentration per trial and the results expressed as mean ± standard deviation (SD). To determine significant differences between the control and each treatment group, the data were subjected to one-way analysis of variance (ANOVA) followed by post-hoc Dunnett’s *t*-test using SPSS Statistics 21 software. Effects were considered statistically significant at *p* ≤ 0.05.

## 3. Results and Discussion

### 3.1. Changes in Growth Profiles of E. gracilis Under Ethanol-Guaiacol Co-Regulation

**Figure 1:**
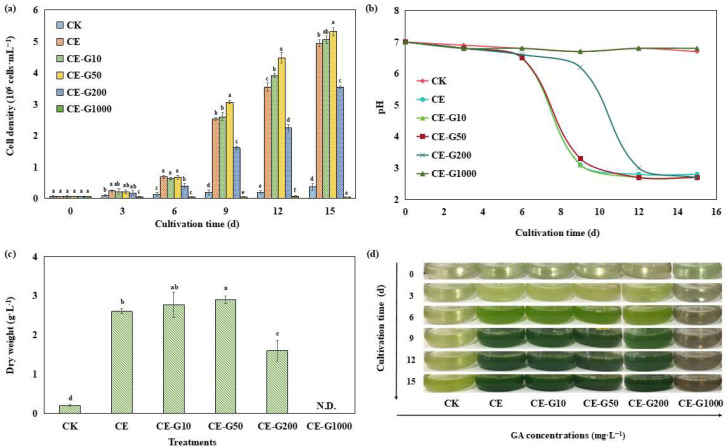
Growth profiles of *E. gracilis* under different treatments. (**a**): cell density; (**b**): filtrate pH; (**c**): DW; (**d**): growth photos. CK: control; CE: solvent control containing 1% ethanol; CE-G10: 10 mg·L^−1^ GA; CE-G50: 50 mg·L^−1^ GA; CE-G200: 200 mg·L^−1^ GA; CE-G1000: 1000 mg·L^−1^ GA. N.D. represents no determination. Different lowercase letters indicate significant differences among groups (*p* < 0.05).

As shown in Figure 1, compared with photoautotrophic culture, 1% ethanol-induced mixotrophy significantly increased the cell density and DW of *E. gracilis* and shortened the growth cycle. At 15 days, the cell density and DW of the CE-treated group reached 13.64-fold and 12.68-fold, respectively, of those of the CK group (*p* < 0.01). Furthermore, the growth of *E. gracilis* under guaiacol (GA) treatment showed a typical bidirectional regulatory effect of promotion at low concentration and inhibition at high concentration. The cell density and DW of cells added with 50 mg·L^−1^ GA increased by 7.47% and 11.54%, respectively, compared with the CE-treated group. When the concentration of GA was increased to 200 mg·L^−1^ (CE-G200), the cell density decreased by 28.35% (*p* < 0.05) compared with the CE group, but was still higher than that of the CK group. In contrast, the cells in the 1000 mg·L^−1^ GA group (CE-G1000) were completely inhibited, exhibiting cell death, rupture, and decoloration. The trend of cell DW change was consistent with the cell density results (Figure 1c).

The pH of the CK-treated group was stabilized at 6.7 ± 0.3 within 15 days, whereas the high-concentration GA group (CE-G1000) was not significantly different from CK due to the complete death of algal cells and stagnation of pH change (*p* > 0.05). In contrast, the pH of the heterotrophic group (CE) containing 1% ethanol and the low-concentration GA group (CE-G10~CE-G200) plummeted to 2.7 within 15 days (Figure 1b), which was mainly attributed to the large amount of CO_2_ produced by the excessively high algal density, which dissolved in the water, leading to the decrease in pH [14]. Crucially, the low-pH environment (pH 2.7) is not toxic but physiologically favorable for *E. gracilis*. As an acidophilic microalga, it maintains intracellular pH homeostasis through vacuolar proton pumps and efficient bicarbonate uptake systems [15], and the low pH can effectively suppresses bacterial growth, which is beneficial to outdoor cultivation [16]. The medium color change was consistent with the growth trend (Figure 1d): the color of the low-concentration GA group (10, 50 mg·L^−1^) deepened from light green to dark green, whereas the high-concentration group (≥200 mg·L^−1^) was lighter in comparison to the low-concentration group, and the CE-G1000 group ended up with a brownish turbid color, which may indicate that high concentrations of GA undergoes autooxidation, which further supports the complete inhibition of algal cell growth at high concentrations.

The concentration-dependent regulation of *E. gracilis* growth by GA (promotion at low concentrations and inhibition at high concentrations) is consistent with the typical stimulatory effects of phenolic compounds. Low concentrations of GA (≤50 mg·L^−1^) may promote proliferation through activation of glycolytic pathways (e.g., up-regulation of ethanol dehydrogenase activity) and scavenging of reactive oxygen species (ROS), which is similar to the growth-promoting phenomenon of organic acid accumulation in yeast heterotrophic metabolism [17]. In contrast, high concentrations of GA (≥200 mg·L^−1^) generate large amounts of ROS through photoauto-oxidation, which inhibits growth by disrupting membrane integrity (e.g., inhibiting ATP synthase activity) and triggering programmed death (e.g., vesicle rupture) [15]. Medium color changes intuitively reflect cellular status: dark green color is associated with enhanced chlorophyll synthesis and healthy proliferation, while brown color suggests cell lysis and accumulation of oxidation products (e.g., quinones) [18]. The color attenuation in the high-concentration GA group coincided with the decrease in cell density, suggesting that light-shielding effects may also be involved in cell growth inhibition. Specifically, high-concentration GA solutions (≥200 mg·L^−1^) exhibit intrinsic brown coloration due to auto-oxidation products (e.g., quinones) [18], which absorb photosynthetically active radiation and attenuate light penetration depth in the culture medium. This reduced light availability compromises photosynthetic efficiency and energy metabolism, thereby synergizing with GA toxicity to suppress cellular proliferation.

Overall, low-dose GA synergized with ethanol treatment significantly increased the biomass of *E. gracilis*, while the excessively low pH environment (pH < 3) was also effective in inhibiting stray bacterial contamination. This dual advantage not only facilitates the stability of outdoor scale cultivation but also provides an innovative solution for the integrated production of microalgae-based functional food ingredients, such as high-protein algal powder or antioxidants [19,20].

### 3.2. Changes in UV-Vis Spectra of the Culture Medium

**Figure 2:**
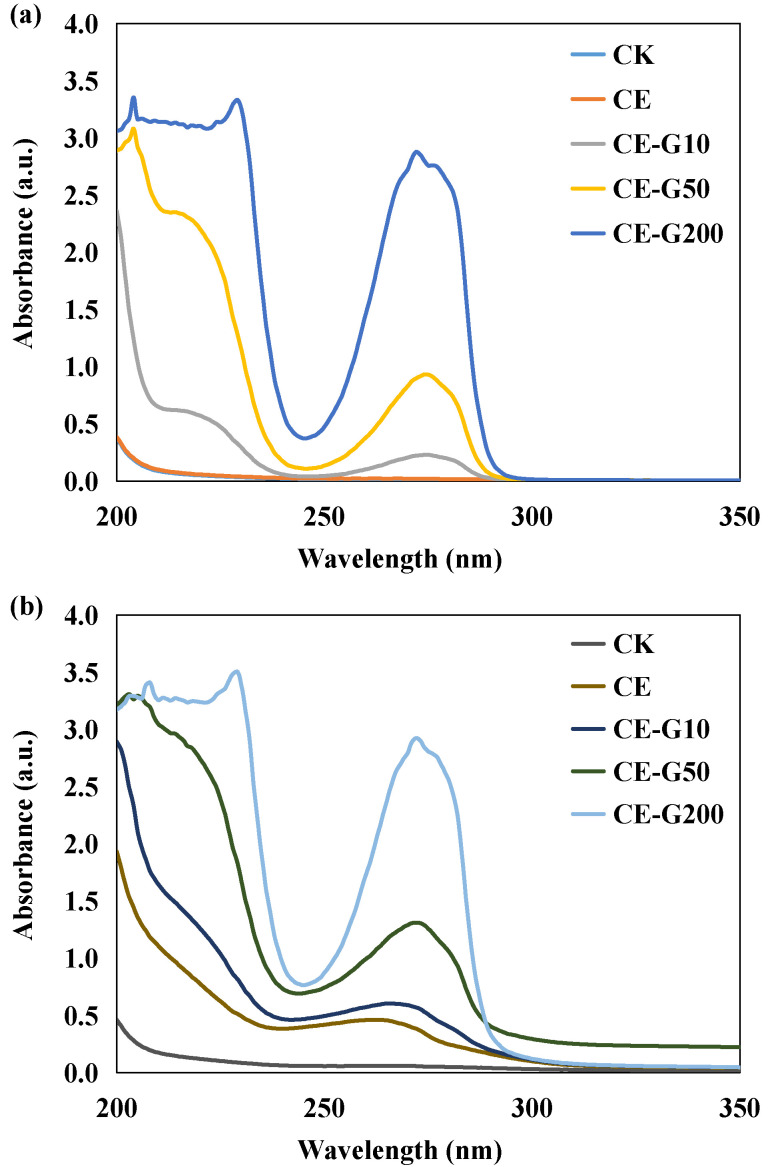
UV-vis spectra of microalgae filtrate at different cultivation times: (**a**) 0 day; (**b**) 15th day. CK: control; CE: solvent control containing 1% ethanol; CE-G10: 10 mg·L^−1^ GA; CE-G50: 50 mg·L^−1^ GA; CE-G200: 200 mg·L^−1^ GA.

The standard curves showed a highly linear correlation (correlation coefficient R^2^ = 0.9933) between GA concentration and absorbance at 275 nm (Abs275), indicating that Abs275 can be used as a reliable quantitative indicator of GA content. At 0 days of incubation (Figure 2a), the deviation of measured and theoretical values of Abs275 from the theoretical values was less than 5% for all GA treatment groups. After 15 days of incubation (Figure 2b), the UV spectra of the GA treatment groups changed significantly: the characteristic absorption peak exhibited varying degrees of blue-shift from the initial 275 nm (Figure S1). This spectral change may be attributed to the auto-oxidation of GA. The hormesis effect of GA may be closely related to the quinones generated by its oxidation. The small amount of quinones generated by the oxidation of low concentrations of GA (≤50 mg·L^−1^) can act as electron acceptors to enhance metabolic activity by promoting NADH regeneration [21,22], thereby stimulating microalgal growth. In contrast, high concentrations of GA (≥200 mg·L^−1^) triggered a burst of ROS and inhibition of enzyme activities (e.g., GA dehydrogenase) due to quinone over-accumulation, leading to lipid peroxidation and a collapse of energy metabolism, similar to the mechanism of oxidative stress in *Chlorella vulgaris* in response to antibiotic exposure [23,24].

### 3.3. Photosynthetic Pigment Analysis

**Figure 3:**
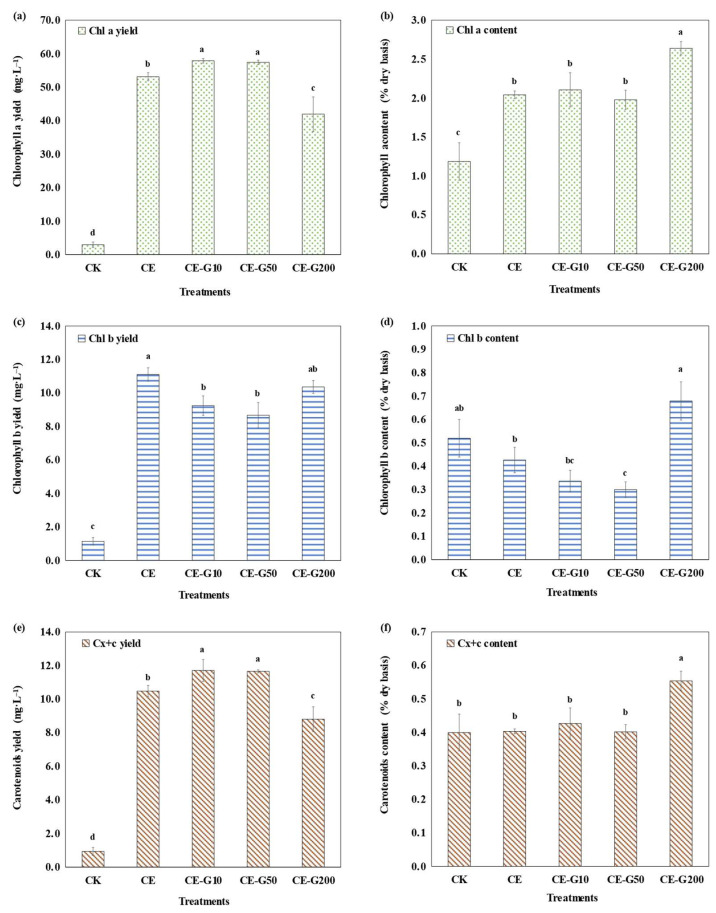
Pigments yield and content of *E. gracilis* under different treatments. (**a**): chlorophyll a yield (mg·L^−1^); (**b**): chlorophyll a content (% dry basis); (**c**): chlorophyll b yield (mg·L^−1^); (**d**): chlorophyll b content (% dry basis); (**e**): carotenoids yield (mg·L^−1^); (**f**): carotenoids content (% dry basis). CK: control; CE: solvent control containing 1% ethanol; CE-G10: 10 mg·L^−1^ GA; CE-G50: 50 mg·L^−1^ GA; CE-G200: 200 mg·L^−1^ GA. Different lowercase letters indicate significant differences among groups (*p* < 0.05).

**Figure 4:**
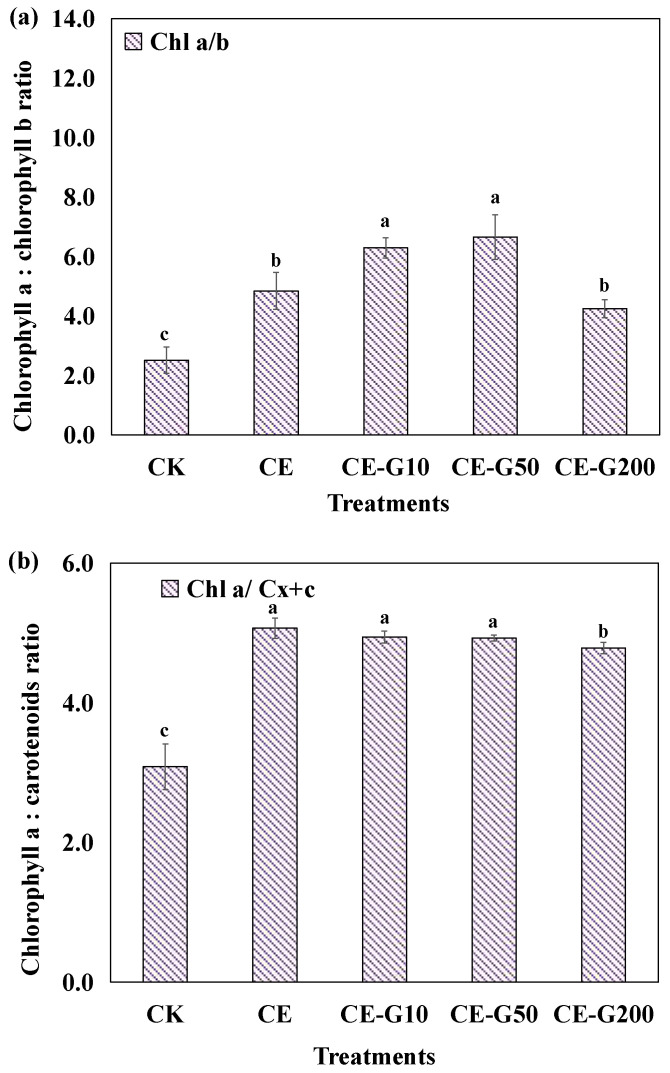
The chlorophyll a/b ratio, and the chlorophyll a/carotenoids ratio in *E. gracilis* under different treatments. (**a**): chlorophyll a to chlorophyll b ratio; (**b**): chlorophyll a to carotenoids. CK: control; CE: solvent control containing 1% ethanol; CE-G10: 10 mg·L^−1^ GA; CE-G50: 50 mg·L^−1^ GA; CE-G200: 200 mg·L^−1^ GA. Different lowercase letters indicate significant differences among groups (*p* < 0.05).

The yields (mg·L^−1^) and contents (% of dry cell weight, denoted as % dry basis) of photosynthetic pigments under different treatments are summarized in Figure 3. The CE-treated group exhibited 18.16-fold, 9.73-fold, and 11.24-fold enhancements in Chl_a_ yield, Chl_b_ yield, and C_x+c_ yield, respectively, compared to the CK group, suggesting that the mixtrotrophic model can significantly increase the production capacity of products such as chlorophylls and carotenoids, laying the foundation for large-scale culture. Both 10 mg·L^−1^ and 50 mg·L^−1^ GA increased the yields of Chl_a_ and C_x+c_ by 8.96% and 8.10%, and 11.75% and 11.23%, respectively, while decreasing the yields of Chl_b_ by 16.80% and 21.93% compared with the CE group. This is consistent with the observed increase in the Chl_a_/Chl_b_ ratio (Figure 4a). This shift suggests preferential resource allocation toward photosystem I core pigments (Chl_a_) over light-harvesting antennae (Chl_b_) to optimize photosynthetic efficiency under chemical elicitation.

When GA concentration reached 200 mg·L^−1^, the yields of Chl_a_ and C_x+c_ decreased by 27.52% and 24.79%, respectively, compared to the peak group. However, changes in photosynthetic pigment content responded differently to GA treatment. The changes in the contents of Chl_a_, Chl_b_, and C_x+c_ were not significant at low concentrations of GA treatment. However, at a GA concentration of 200 mg·L^−1^, the dry basis contents of Chl_a_, Chl_b_, and C_x+c_ increased by 29.30%, 59.29%, and 37.34%, respectively, as compared to the CE group. It is important to note that the quantification of photosynthetic pigments in this study was performed using spectrophotometric methods. It provides an approximate determination of pigment concentrations, but lacks the resolution of HPLC for specific carotenoid isomers. However, this method is validated for detecting relative changes in comparative studies, and the consistent methodology across all treatments ensures the robustness of observed trends.

To further understand their effects on the photosynthetic capacity and physiological state of algal cells, changes in the ratio of Chl_a_ to Chl_b_, and the ratio of Chl_a_ to C_x+c_ were analyzed. The Chl_a_/Chl_b_ ratio serves as a key indicator of the relative size and composition of the light-harvesting antennae associated with Photosystem II (PSII) versus the core reaction centers [25]. Chl_a_ is the primary pigment in both PSI and PSII reaction centers, while Chl_b_ is predominantly found in the light-harvesting complexes (LHCs), especially those associated with PSII [25,26]. An increase in the Chl_a_/Chl_b_ ratio typically signifies a reduction in the size of the peripheral LHCs relative to the core complexes, optimizing light utilization efficiency under conditions favoring high photosynthetic rates or high light. Conversely, a decrease in this ratio often indicates an expansion of the LHC antennae to enhance light capture under low-light conditions [27,28]. The Chl_a_/C_x+c_ ratio reflects the balance between light-harvesting/energy conversion capacity (primarily driven by chlorophylls) and photoprotective/antioxidant capacity (provided by C_x+c_). A decrease in this ratio often signals increased cellular stress, prompting greater investment in carotenoids for quenching excess excitation energy and scavenging ROS [29,30]. The ratio of Chl_a_ to Chl_b_ ratio was significantly higher in the CE group compared with the CK, and the treatment of low concentrations of GA (10, 50 mg·L^−1^) further increased the ratio, indicating that the low concentration of GA optimized the photosystem II activity by enhancing the light capture capacity [28]. The ratio significantly decreased below the CE level under 200 mg·L^−1^ GA treatment, which was consistent with the increasing trend of Chl_b_ content. This suggests that the high GA concentration contributed to an increased photosystem II to photosystem I ratio, allowing cells to adapt to a low-light environment through a shading effect [31]. The ratio of Chl_a_ to C_x+c_ reached its maximum value in the CE group. The low-concentration GA treatment groups (10, 50 mg·L^−1^) were slightly lower than CE but still in the physiologically healthy range (4–6) [29], suggesting that ethanol and low-concentration GA synergistically maintained the balance between light energy conversion efficiency and antioxidant capacity. However, the 200 mg·L^−1^ GA treatment resulted in a significant decrease in the ratio of Chl_a_ to C_x+c_, indicating that the physiological status of the cells was poor, which was mainly due to the decrease of Chl_a_ content and the increase of C_x+c_ content. This is a common feature of pigment changes in microalgae under strong light or nitrogen deficiency stress [30]. This mode of regulation may enhance the survival adaptability of microalgae under high-stress environments by optimizing photosynthetic efficiency and antioxidant capacity.

### 3.4. Analysis of Paramylon Production

**Figure 5:**
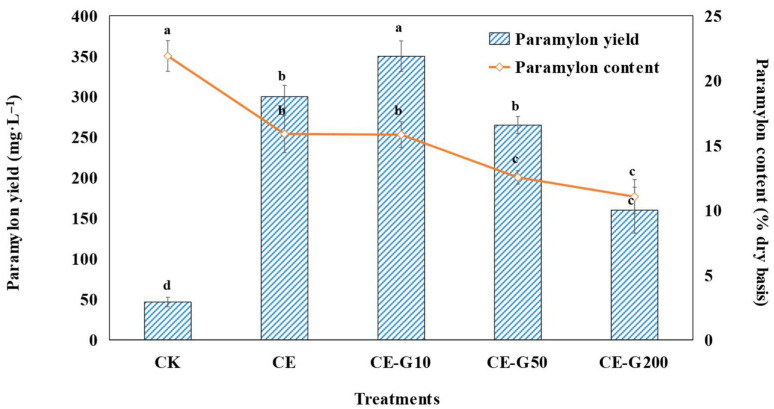
Paramylon yield and content of *E. gracilis* under different treatments. CK: control; CE: solvent control containing 1% ethanol; CE-G10: 10 mg·L^−1^ GA; CE-G50: 50 mg·L^−1^ GA; CE-G200: 200 mg·L^−1^ GA. Different lowercase letters indicate significant differences among groups (*p* < 0.05).

As summarized in Figure 5, ethanol-induced mixotrophy triggered a metabolic shift in *E. gracilis*, favoring biomass proliferation over paramylon accumulation. While total biomass surged 12.68-fold, cellular paramylon content decreased by 27.46% compared to photoautotrophic controls (CK), resulting in a net 6.43-fold yield gain (Figure 5). This discrepancy arises because organic carbon (ethanol) supplies abundant acetyl-CoA and ATP, which are preferentially channeled toward rapid cell division, membrane synthesis, and protein production rather than storage glucans [32]. Consequently, although paramylon yield increases absolutely due to massive biomass expansion, its cellular content declines—a trade-off characteristic of mixotrophic microalgae leveraging organic carbon for growth acceleration [10,32]. GA further modulates this balance: at 10 mg·L^−1^, it enhances the paramylon yield by 16.67% without compromising growth, likely by fine-tuning carbon partitioning toward glucan polymerization. Specifically, the CE group exhibited a 6.43-fold yield increase over CK (*p* < 0.01), which was further elevated by 16.67% (*p* < 0.05) upon 10 mg·L^−1^ GA addition (7.50-fold vs. CK; 1.17-fold vs. CE). However, higher GA concentrations reversed this trend, with 50 mg·L^−1^ and 200 mg·L^−1^ GA groups showing 24.29% and 46.67% yield reductions, respectively, compared to their optimal predecessors, mirroring biomass DW dynamics.

This inverse relationship between paramylon content and total yield suggests metabolic prioritization: cells under chemical stress preferentially allocate energy towards proliferation rather than polysaccharide synthesis. Notably, our optimal ethanol-GA co-regulation strategy (CE-G10 group) achieved a paramylon yield increase of 7.50-fold over the autotrophic CK. When compared with recent industrial byproduct-based cultivation systems, our approach demonstrates comparable potential. Kim et al. [33] reported paramylon yields of 0.25–1.80 g·L^−1^ using corn steep liquor (CSL) as sole substrate, which corresponds to 16.90–54.2% of dry cell weight (DCW). Our CE-G10 system achieved 0.35 g·L^−1^ paramylon with 15.82% DCW content—comparable to Kim et al.’s CSL system but without requiring specialized industrial byproducts. Furthermore, our observed reduction in cellular paramylon content under ethanol mixotrophy (27.46% decrease vs. CK) aligns with reports that organic carbon sources redirect metabolism toward growth rather than storage polymer synthesis [32]. The 16.67% yield enhancement with low-dose GA (10 mg·L^−1^) is particularly significant as it demonstrates the potential of phenolic elicitors to further boost productivity beyond standard mixotrophic cultivation.

The achieved yield enhancement holds industrial significance. As a functionally validated β-1,3-glucan with immunomodulatory and prebiotic properties [4,6], paramylon’s scalable production through this strategy improves the economic viability of functional food development, demonstrating efficient resource utilization in algal biotechnology. The combination of high volumetric yield, acidophilic cultivation that minimizes contamination risks, and use of low-cost biomass-derived stimulants (ethanol and GA) presents a compelling case for commercial scale-up.

## 4. Conclusions

In conclusion, this study demonstrates that ethanol-guaiacol co-regulation synergistically enhances paramylon biosynthesis in *E. gracilis* through a biphasic hormetic mechanism. Ethanol-induced mixotrophy elevated biomass and paramylon yields by 12.68-fold and 6.43-fold, respectively, compared to photoautotrophic controls. Low-dose GA (10 mg·L^−1^) further amplified paramylon productivity by 16.67% via optimized carbon partitioning, while concurrently boosting Chl_a_ (+8.96%) and C_x+c_ (+11.75%) synthesis. Conversely, GA concentrations ≥200 mg·L^−1^ suppressed growth due to quinone-mediated ROS generation and light attenuation, confirming its dose-dependent dual role. Notably, the self-acidified low-pH environment (pH~2.7) inherent to this mixotrophic system effectively minimized bacterial contamination, enhancing scalability for outdoor cultivation. As the first report of GA-mediated hormesis in *E. gracilis*, this strategy leverages low-cost biomass-derived stimulants (ethanol and GA) to advance sustainable microalgal biorefineries. Future work should prioritize elucidating GA’s transmembrane transport dynamics, transcriptional regulation of β-1,3-glucan synthase, and techno-economic optimization of integrated phenolic-ethanol elicitation platforms for industrial translation.

## Data Availability

Data will be made available on request.

## Supplementary Materials

The following supporting information can be downloaded at: https://www.mdpi.com/article/10.3390/foods14142457/s1, Figure S1. UV-vis Spectra of Microalgae Filtrate in CE-G10 Treatment Group at Different Time Points.
