# Supplementary material for: Synergistic Enhancement of Paramylon Production in Edible Microalga Euglena gracilis via Ethanol-Guaiacol Co-Regulation
Source: Foods. 2025 Jul 12;14(14):2457. doi: 10.3390/foods14142457 (PMC12294735; doi:10.3390/foods14142457)
Supplement: Supplementary file 1 [file foods-14-02457-s001.zip › foods-3690071-supplementary.pdf]

Supplementary File

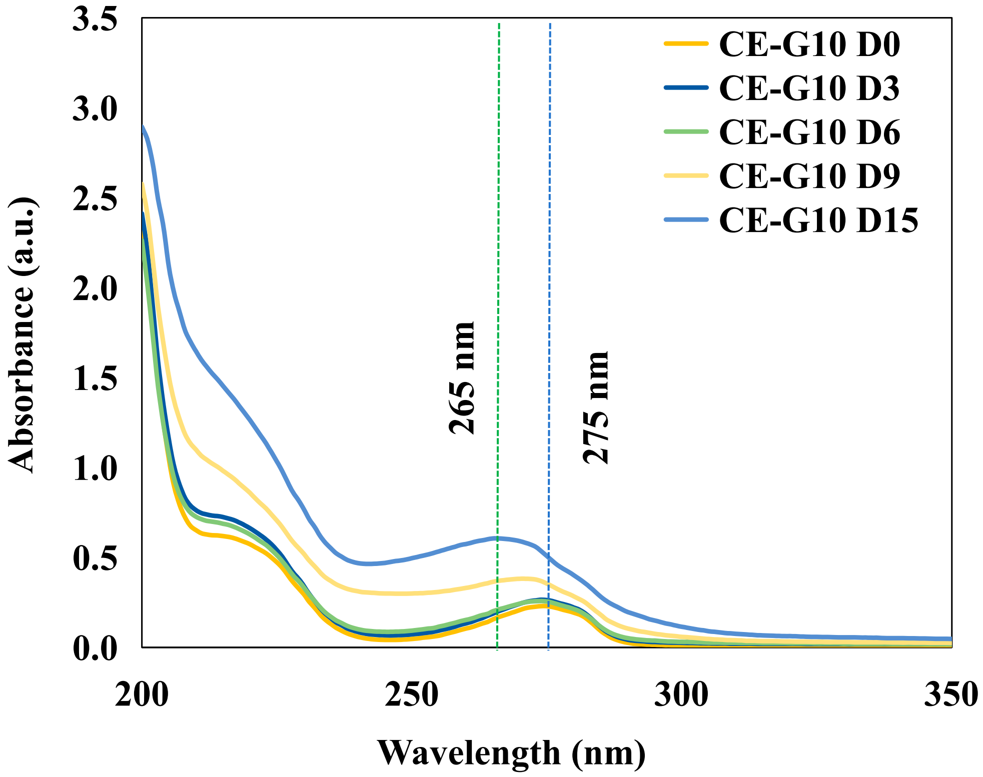

Figure S1 UV-vis Spectra of Microalgae Filtrate in CE-G10 Treatment Group at Different Time Points.
